# Skeletal Muscle Ultrasonography and Muscle Fitness Relationships: Effects of Scanning Plane and Echogenicity Correction

**DOI:** 10.3390/muscles2020010

**Published:** 2023-03-23

**Authors:** Caleb Voskuil, Monique Dudar, Yan Zhang, Joshua Carr

**Affiliations:** 1 Department of Kinesiology, Texas Christian University, Fort Worth, TX 76129, USA; 2 Harris College of Nursing & Health Sciences, Texas Christian University, Fort Worth, TX 76109, USA; 3 Department of Medical Education, Texas Christian University School of Medicine, Fort Worth, TX 76129, USA

**Keywords:** resistance training, muscle strength, muscle quality, muscle endurance

## Abstract

This study examines the relationships between ultrasonography measurements of skeletal muscle size and echo intensity (EI) with muscle strength and local muscle endurance in a habitually resistance-trained population. Twenty young, healthy participants underwent imaging of the biceps brachii in the sagittal and transverse planes and with the extended field of view (EFOV) technique. Linear regression was used to examine measures of muscle thickness (MT), muscle cross-sectional area (mCSA), EI, and corrected EI (cEI) in each scanning plane for their associations with strength (1RM biceps curl) and local muscle endurance (4x failure @ 50%1RM). The strongest predictor of 1RM strength and local muscle endurance was sagittal MT (adj. R^2^ = 0.682) and sagittal cEI (adj. R^2^ = 0.449), respectively. Strength and transverse MT (R^2^ = 0.661) and the EFOV mCSA (R^2^ = 0.643) demonstrated a positive relationship. Local muscle endurance and cEI in the transverse plane (R^2^ = 0.265) and the EFOV scan (R^2^ = 0.309) demonstrated a negative relationship. No associations were shown with uncorrected EI. While each scanning plane supports the muscle size-strength and echogenicity-endurance relationships, sagittal plane imaging demonstrated the strongest associations with muscle fitness. These findings provide important methodological insights regarding ultrasound imaging and muscle fitness relationships.

## 1. Introduction

The use of B-mode ultrasonography for skeletal muscle imaging is growing in popularity in the fields of sports medicine and allied health due to its increasing accessibility [1,2,3]. Compared to the other imaging techniques like magnetic resonance imaging (MRI), computed tomography (CT), and dual-energy *x*-ray absorptiometry (DXA), ultrasonography provides measures of skeletal muscle size and echogenicity in a fraction of the time and cost, while producing reliable and comparable results [3,4,5,6]. B-mode ultrasonography imaging is commonly performed in the sagittal or the transverse planes with the images being captured in a still frame or with the extended field of view technique (EFOV) where multiple frames are stitched together with specialized software [4,7,8,9,10]. Muscle size can be quantified as a linear measure of muscle thickness in the sagittal and transverse planes [8,11], or with muscle cross-sectional area utilizing the panoramic image obtained with EFOV [7,10]. More recently, the echo intensity (EI) of skeletal muscle images has been used as a loose reference for muscle quality [3]. The echogenicity of the skeletal muscle shows peculiar relationships with muscle glycogen levels [12,13,14,15], clinical measures [16,17,18,19,20], and muscle function [21,22,23,24]. However, the interpretations of EI as a measure of muscle quality are not unanimous [3]. Although ultrasound-derived measures of muscle morphology are valid and demonstrate acceptable reliability [4], subtle methodological factors regarding image acquisition can affect the outcomes [11,25]. Given the importance of muscle size and echogenicity measurements in practical and clinical settings, identifying how different scanning methods relate to muscle fitness parameters may offer directions in time-restricted settings.

Skeletal muscle size is a known determinant of muscle strength [26]. However, the association between ultrasonography-derived skeletal muscle morphology measurements and muscle fitness is not well described [3]. Muscle thickness, cross-sectional area, and their regionalization along the limb all generally show moderate to strong positive relationships with muscle strength [27]. However, EI has received much less focus, namely due to conflicting findings and disagreements on application [3]. Echo intensity has shown weak to moderate associations with strength and agility in adolescents [21,28], gait speed and activities of daily living in older adults [23,29], and occupational and sport-related tasks in healthy adults [30,31]. The association between EI and gait speed and other functional tasks [23,29,30,31] suggests that EI may be related to local muscle endurance. However, there are extremely limited data regarding the relationships between these skeletal muscle outcomes and muscle fitness in habitually resistance-trained populations. It may be that methodological considerations regarding image acquisition as well as corrections for adipose tissue thickness [3,6,23,29] have an influence on the discrepancies seen in the literature. Greater insight into how the various techniques (i.e., transverse MT, sagittal EI) affect the relationships between ultrasonography-derived outcomes and muscle fitness will offer important insights.

Therefore, the purpose of this study is to (1) identify the associations between ultrasound-derived measurements of skeletal muscle morphology with muscle fitness during the biceps curl in a habitually resistance-trained population, and (2) determine the technique with the strongest association with maximal strength and local muscle endurance. The maximal strength and local muscle endurance of the biceps brachii quantified muscle fitness during the biceps curl exercise. Ultrasonography images of the biceps brachii were collected in the sagittal and transverse planes, along with the EFOV technique. The measurements of muscle thickness, cross-sectional area, EI, and echo intensity corrected for adipose tissue thickness (cEI) were used to quantify skeletal muscle morphology. Each of these measures were examined for their association with muscular strength and endurance and run through multiple linear regression.

## 2. Results

### 2.1. Correlations

The associations between skeletal muscle ultrasonography outcomes with maximal strength and local muscle endurance are reported in Table 1.

### 2.2. Stepwise Multiple Linear Regression

**Figure 1:**
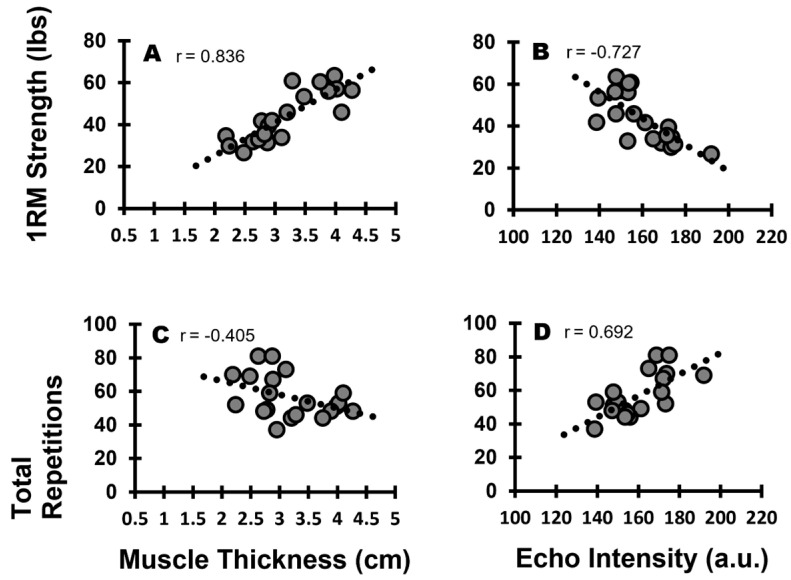
The relationships between biceps brachii ultrasonography outcomes with maximal strength (**A**,**B**) and local muscle endurance (**C**,**D**) in the sagittal plane. The strongest predictor of maximal strength was sagittal plane muscle thickness (**A**), whereas the strongest predictor of local muscle endurance was sagittal plane corrected EI (**D**).

Stepwise multiple linear regression was run to assess if three ultrasound measures (muscle size, EI, and cEI) in three different scanning planes (sagittal, transverse, and EFOV) can predict maximal strength (1RM) and local muscle endurance (total repetitions) in the biceps curl. The regression model indicated that muscle thickness in the sagittal plane was the strongest predictor of 1RM strength (F(1,18) = 41.742, *p* < 0.001, adjusted R^2^ = 0.682; SEE = 6.74 lbs). Meanwhile, the model for local muscle endurance indicated that cEI in the sagittal plane was the strongest predictor of total repetitions (F(1,18) = 16.512, *p* < 0.001, adjusted R^2^ = 0.449; SEE = 9.45 repetitions). Figure 1 shows the relationships between the sagittal plane ultrasonography outcomes with muscle strength (Figure 1A,B) and muscle endurance (Figure 1C,D). The prediction models are shown in Supplemental File S1.

## 3. Discussion

This study examined the relationships between skeletal muscle ultrasound outcome variables with muscle fitness during the biceps curl in a habitually resistance-trained population. The main findings show that (1) sagittal plane imaging of muscle thickness and echogenicity demonstrated the strongest associations with 1RM strength and with local muscle endurance, respectively; (2) there was a strong, positive relationship between muscle size and biceps curl 1RM for all imaging techniques; and (3) the echogenicity of the biceps brachii showed moderate to strong associations [32] with maximal strength and local muscle endurance, but only when corrections for subcutaneous adipose tissue thickness were applied. Overall, we showed that muscle thickness obtained in the sagittal plane is the strongest predictor of biceps curl 1RM strength, while the EI derived from the sagittal plane demonstrated the greatest association with local muscle endurance. Interestingly, uncorrected EI did not show significant associations with biceps curl 1RM or local muscle endurance as recent findings have shown [22,33,34]. While each ultrasonography imaging plane showed significant relationships with muscle fitness, our findings suggest that images obtained in the sagittal plane sufficiently explain these relationships. These findings also highlight the importance of correcting EI for adipose tissue thickness to increase validity and aid in the translation of EI as a muscle fitness predictor in resistance-trained populations.

The muscle size–strength relationship is well-documented for large muscles of the upper and lower limbs [21,28,33,35,36]. Dynamic muscle strength and muscle size demonstrate a positive relationship in both single- [33] and multi-joint [36] movements. Back squat 1RM strength demonstrates moderate to strong associations with transverse MT (R = 0.56) and mCSA (R = 0.60) of the vastus lateralis in trained participants [36]. Single-joint movements like biceps curl (R = 0.899–0.905) and knee extension (R = 0.695–0.676) 1RM demonstrate strong associations with sagittal MT in a population of trained and untrained individuals [33]. The current study reports similar findings across the sagittal and transverse plane, and with the EFOV technique in a habitually resistance-trained population performing a single-joint movement. As multiple synergistic muscles contribute to back squat or leg extension 1RM, their muscle size–strength relationships are weaker than those observed in biceps curls, in which the biceps brachii is the prime mover. Examinations of the relationship between EI and muscle strength are much more scarce [21,29,34]. Isokinetic knee extension strength demonstrates a negative relationship with uncorrected EI in the elderly (R = −0.333) and corrected EI in adolescents (R = −0.241–−0.524) [21,28,34]. The relationship between EI and strength may be stronger during higher velocity movements following a correction for adipose tissue thickness [28]. The current study did not observe a relationship between uncorrected EI and strength and supports this methodological consideration. While the impact of resistance training protocols (<1 year) on EI has demonstrated conflicting results [4,8,22,37], the resistance training experience ($\bar{x}$ = 4 years) of the participants in the current study may have altered skeletal muscle echogenicity and strengthened the inverse relationship with maximal strength. This experiment demonstrated that muscle size and echogenicity have a strong to moderate correlation with 1RM strength in a resistance-trained population. Additionally, the sample consisted of an equal number of resistance-trained males and females, providing generalizability for a commonly performed dynamic resistance training exercise. These findings support the use of ultrasonography as a muscle strength predictor in sports medicine and allied health settings and for populations having undergone chronic resistance training.

As described previously, the interpretations that stem from EI measurement outcomes have not reached a consensus due to the variety of factors that influence the measurements [3]. The current study demonstrated the importance of adipose tissue correction of echo intensity, as no relationship with muscle fitness was shown with uncorrected EI. However, the cEI demonstrated strong to moderate positive associations with local muscle endurance. The relationship between local muscle endurance and EI observed in the current study has not been noted previously, as examinations of knee extensions to failure and incremental testing on a cycle ergometer reported no associations with EI [22,23]. The knee extensions performed to failure were at a similar intensity (50% MVC) as the current study and utilized the same procedure to correct for adipose tissue thickness but reported no relationship with EI [23]. The differences in findings in the current study may relate to the inclusion of habitually resistance-trained individuals, rather than older or untrained populations. Age-related increases in intramuscular tissue and training-related increases in capillarization may explain these differences, but further research is recommended to clarify this relationship [22,23]. Interestingly, MT and local muscle endurance showed a strong to moderate negative relationship. While increases in MT may aid the force-generating capacity of a muscle, an increase in MT may increase the intramuscular pressure within the muscle and reduce the clearance of nociceptive metabolites [38,39,40,41]. Muscle fiber type may influence this relationship, as type I muscle fibers demonstrate a greater oxidative capacity and greater intramuscular fat and lipid content [42,43]. Type II muscle fibers may produce a relatively greater number of metabolites than type I muscle fibers, reducing the fatigue capacity of a muscle with greater type II muscle content [38,39,40,41]. As a greater EI value is hypothesized to represent a greater amount of intramuscular fat [3,6,44], a muscle with a larger amount of type I muscle fibers may present with a greater echo intensity. Muscle biopsy work demonstrates intramuscular lipid content has a strong to moderate positive relationship with echo intensity, suggesting that this measure may be utilized as a cost-effective and non-invasive method to examine body composition [6,45]. The strong to moderate relationships between local muscle endurance and cEI in the current study further supports the use of echogenicity as a muscle fitness predictor.

There are some important limitations to note regarding the findings of this study. It has been shown that skin color influences EI [46,47,48]. The participants that volunteered for this study were of Caucasian, Hispanic, and Asian descent, and had similar skin pigmentation. This important methodological note should be considered when comparing EI across populations and examined in future studies. As the average age of this population was ~21 years of age, it is important to consider the age-related increases in fibrous tissue within the muscle that may influence EI when generalizing this data to elderly populations [3,22]. Additionally, the sample size for the current study indicated adequate power for the correlations but is insufficient for the regression predictions. Due to the sample size, the findings of the regression predictions should be interpreted with caution. In the current study, only the biceps brachii was imaged and analyzed for MT and echo intensity. As the biceps brachii is the prime mover of the elbow, the ultrasound-derived measures of this muscle alone may be sufficient to examine the relationship of MT and EI with biceps curl muscle fitness. However, if muscle fitness in multi-joint or single-joint movements with multiple synergist muscles are examined, ultrasound imaging of all contributing muscles is suggested. Lastly, we are unable to determine whether ultrasound image acquisition or analyses explains our findings as each may uniquely influence image outcomes given the variability contained within these two separate steps [11]. These limitations provide opportunities for further research and caution when generalizing the findings reported in the current study.

This experiment examines important methodological considerations for ultrasonography imaging for inferences regarding muscle fitness. Our data showed that ultrasonography imaging of the biceps brachii across the three common scanning planes provides morphology outcomes (i.e., MT and EI) that associate with muscle fitness. Importantly, our data suggest that sagittal plane imaging has the strongest associations with muscle fitness compared to the transverse and EFOV measures. Our data support other recent findings showing the importance of correcting for subcutaneous thickness for interpreting EI [3,6,29,49]. Overall, these findings suggest sagittal plane imaging is likely the preferred scanning plane for the biceps brachii. These findings are useful for time-restricted scenarios that are common in laboratory, sports medicine, and allied health settings. Additionally, correcting EI for adipose tissue thickness should be performed to increase the validity of the EI measure as an indicator of muscle quality. These data present methodological considerations that may optimize ultrasonography imaging practices while supporting future applications of echo intensity.

## 4. Materials and Methods

### 4.1. Experimental Design

This study used a cross-sectional design to identify the associations between ultrasound-derived measurements of skeletal muscle morphology with maximal strength and local muscle endurance during the biceps curl in a habitually resistance-trained population. The study required a total of two visits to the Neuromuscular Physiology Laboratory in the Kinesiology Department at Texas Christian University. The visits were separated by at least 48 h. The first visit included ultrasound imaging and biceps curl 1 repetition maximum strength testing. During the second visit, participants completed a fatiguing resistance exercise protocol that consisted of four sets of biceps curls to failure with their dominant arm.

### 4.2. Participants

A total of 20 participants (females: n = 10, age: 21.3 ± 1.64 years, height: 166.88 ± 4.95 cm, mass: 71.30 ± 6.30 kg; males = 10, age: 21.9 ± 2.51 years, height: 175.51 ± 7.92 cm, weight: 81.47 ± 3.59 kg) were enrolled with all completing the study in its entirety. To qualify for inclusion, participants must have reported ongoing participation in a structured and progressive resistance training program at least 2×/week for the past 6 months. The average training experience reported for females was 4.8 ± 2.2 years at a frequency of 7.5 ± 3.4 h/week, and the males reported 4.6 ± 1.6 years of training experience at a frequency of 7.1 ± 2.5 h/week. The resistance training experience demonstrated by each participant qualified as habitual training, defined as following a structured exercise protocol for an extended period of time. The participant sample included individuals who competed in national weightlifting and bodybuilding events as well as collegiate athletics. Five female participants reported hormonal contraceptive use, while the others indicated eumenorrhea. The female participants not on hormonal contraceptives completed testing during their mid-luteal phase, around day 21 of their menstrual cycle (±2 days); however, one participant completed testing during the late follicular phase due to unforeseen scheduling conflicts. All procedures were approved by the Institutional Review Board for Human Subjects of Texas Christian University (IRB# 2021-222).

### 4.3. Experimental Procedures

#### 4.3.1. Ultrasonography

**Figure 2:**
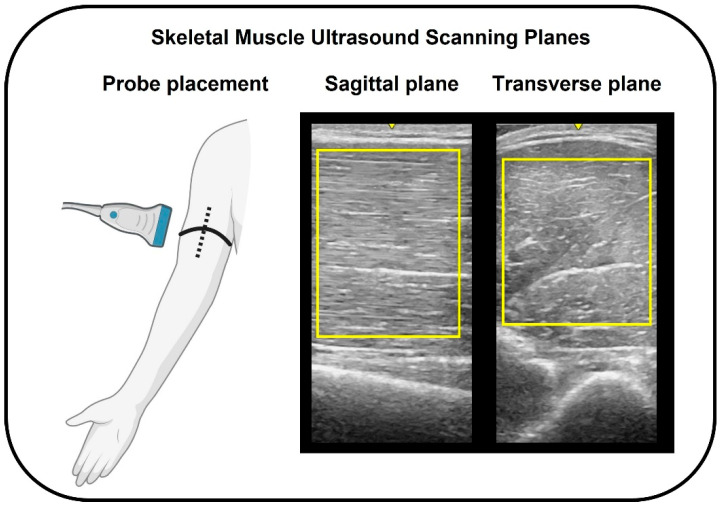
An illustration of the probe placement on the arm and representative ultrasonography images of the biceps brachii. The regions of interest for determining EI (yellow squares, **right** image) for the sagittal (dashed line, **left** image) and transverse plane (solid line, **left** image) images are shown.

Prior to strength testing, B-mode ultrasonography (GE LOGIQ E10; Software Version: R9.1.2; GE Healthcare, Milwaukee, WI, USA) was used to measure muscle size and EI of the biceps brachii of the participant’s dominant arm. The ultrasonography images were collected with a wideband linear array probe (GE L8-18i-RS, 4.5–18 MHz, 25 mm field of view; GE Healthcare, Milwaukee, WI, USA). The settings for the ultrasound were held consistent (Frequency: 12 Hz, Gain: 55 dB, Dynamic range: 75) between each participant, with changes in the depth being made only to accommodate larger muscle size and prevent image overlay in highly curved regions [4,11]. During image acquisition, participants were laying supine on an imaging table with their arm abducted and their palm supinated. Images were taken at 50% of the distance between medial acromion process and the fossa cubit [11] and a generous amount of water-soluble transmission gel was applied to the skin to enhance imaging quality. Images were taken in the sagittal and transverse plane in addition to a panoramic sweep with the EFOV technique until three scans of each met acceptable imaging quality. Of these scans, the highest quality image was chosen for subsequent analyses utilizing ImageJ Software (Version 1.53k). Files were exported following image acquisition to a portable storage device and analyzed as a JPG image. Before analyzing the images, the researchers underwent training to minimize the influence of differences in image analysis experience [11]. Image analysis was performed by first scaling the pixels to cm using the straight-line function. In the sagittal and transverse planes, muscle thickness was determined using the straight-line function at the midpoint of the muscle on a freeze-frame image. Muscle cross-sectional area (cm^2^) was quantified by utilizing the polygon function and outlining the border of each muscle without including the surrounding fascia tissue. EI was determined using the grey-scale analysis histogram function within the same polygon created for cross-sectional area measurements [11]. Echo intensity in freeze-frame sagittal and transverse plane images was determined by using the rectangular function. A maximal rectangular region of interest was created including as much biceps brachii as possible without including the surrounding fascia for each image [11]. Figure 2 provides a representation of the probe placement on the arm and the biceps brachii region of interest determination for the sagittal and transverse plane images.

Measurements of EI were corrected for adipose tissue according to the following equation [6]:

(1) $$\mathrm{Corrected}\;\mathrm{EI}\;=\;\mathrm{Raw}\;\mathrm{EI}\;+\left(\mathrm{Subcutaneous}\;\mathrm{Fat}\;\mathrm{Thickness}\;*\;40.5278\right)$$

#### 4.3.2. Dynamic Strength Testing

During the first visit, the participants performed a dynamic strength assessment of their dominant arm to determine their biceps curl 1RM with an adjustable dumbbell. One repetition maximum testing was performed according to the ASCM guidelines for strength testing [50]. The 1RM was determined in no more than five maximal attempts.

#### 4.3.3. Resistance Exercise Protocol

On the second visit, the participants were required to perform four sets of biceps curls to failure at 50% of their measured 1RM. The participants were seated and asked to perform the repetitions at a controlled pace (50 bpm on a metronome). Participants were required to perform each repetition through the concentric and eccentric phases. Once the participant could no longer perform the movement through the entire range of motion the set was ended. Each set was separated by 2 minutes and the total number of repetitions performed across each set was summed to create the outcome variable of total repetitions.

#### 4.3.4. Statistical Analysis

SPSS (Version 28; IBM Corp., Armonk, NY, USA) was used for all analyses. Frequencies, mean, and standard deviation were obtained to describe the maximal strength and ultrasound-derived measurements of muscle size. Bivariate tests, i.e., Pearson Correlation, examined the relationships between maximal strength and ultrasound-derived measurements of muscle size. Multiple linear regression was run to examine the ultrasound variable that demonstrated the greatest association with maximum strength and local muscle endurance. Nine independent variables from three ultrasound planes were entered stepwise into the model. These included the sagittal plane (muscle thickness, echo intensity, and corrected echo intensity), transverse plane (muscle thickness, echo intensity, and corrected echo intensity), and the extended field of view technique (muscle cross-sectional area, echo intensity, and corrected echo intensity). The modeling was utilized to generate a prediction equation for the 1 repetition maximum and total repetitions. For the 1 repetition maximum strength, an independence of residuals was observed using a Durbin–Watson statistic of 1.678. Linearity and homoscedasticity were observed and demonstrated through a visual inspection of a plot of studentized residuals versus unstandardized predicted values. Normality was met following a histogram plot of standardized residuals. An examination of the multicollinearity demonstrated a violation between sagittal muscle thickness and transverse muscle thickness (r = 0.869) and the EFOV muscle cross-sectional area (r = 0.844). The variable with the greatest correlation with 1RM strength, sagittal muscle thickness, was retained for analysis. No significant outliers were detected by examining the studentized deleted residual and Cook’s Distance at a level of +/− 3 standard deviations. For total repetitions, an independence of residuals was observed by a Durbin–Watson statistic of 2.262. Linearity and homoscedasticity were observed and demonstrated through a visual inspection of a plot of studentized residuals versus unstandardized predicted values. Normality was met following a histogram plot of standardized residuals. An examination of the multicollinearity demonstrated a violation between sagittal cEI and transverse cEI (r = 0.817) and the EFOV cEI (r = 0.771). The variable with the greatest correlation with total repetitions, sagittal cEI, was retained for analysis. No significant outliers were detected by examining the studentized deleted residual and Cook’s Distance at a level of +/− 3 standard deviations.

## Figures and Tables

**Table 1:** Correlation of Skeletal Muscle Ultrasonography Measures with Maximal Strength and Local Muscle Endurance.

	1 Repetition Maximum		Total Repetitions	
Ultrasonography Measure	Pearson Correlation	*p*-Value	Pearson Correlation	*p*-Value
Sagittal View				
Muscle Thickness	0.836	<0.001 *	−0.405	0.038 *
Echo Intensity	−0.350	0.065	0.308	0.093
Corrected EI	−0.727	<0.001 *	0.692	<0.001 *
Transverse View				
Muscle Thickness	0.813	<0.001 *	−0.435	0.028 *
Echo Intensity	−0.230	0.165	0.111	0.321
Corrected EI	−0.618	0.002 *	0.515	0.010 *
Extended Field of View				
Cross-Sectional Area	0.802	<0.001 *	−0.433	0.028 *
Echo Intensity	−0.108	0.325	0.196	0.204
Corrected EI	−0.519	0.01 *	0.556	0.005 *

* Denotes significant correlation at *p* < 0.05 level.

## Data Availability

All data is stored on a public repository with access through the corresponding author upon request.

## Supplementary Materials

The following supporting information can be downloaded at: https://www.mdpi.com/article/10.3390/muscles2020010/s1. Table S1: Prediction Model for 1RM, Table S2: Prediction Model for Total Repetitions.

## References

[1] Fujiwara K., Asai H., Toyama H., Kunita K., Yaguchi C., Kiyota N., Tomita H., Jacobs J.V. (2010). Changes in muscle thickness of gastrocnemius and soleus associated with age and sex. Aging Clin. Exp. Res..

[2] Muraki S., Fukumoto K., Fukuda O. (2013). Prediction of the Muscle Strength by the Muscle Thickness and Hardness Using Ultrasound Muscle Hardness Meter. SpringerPlus.

[3] Stock M.S., Thompson B.J. (2020). Echo intensity as an indicator of skeletal muscle quality: Applications, methodology, and future directions. Eur. J. Appl. Physiol..

[4] Jenkins N.D.M., Miller J.M., Buckner S.L., Cochrane K.C., Bergstrom H.C., Hill E.C., Smith C.M., Housh T.J., Cramer J.T. (2015). Test-Retest Reliability of Single Transverse versus Panoramic Ultrasound Imaging for Muscle Size and Echo Intensity of the Biceps Brachii. Ultrasound Med. Biol..

[5] Nijholt W., Scafoglieri A., Jager-Wittenaar H., Hobbelen J.S.M., van der Schans C.P. (2017). The Reliability and Validity of Ultrasound to Quantify Muscles in Older Adults: A Systematic Review. J. Cachex-Sarcopenia Muscle.

[6] Young H.-J., Jenkins N.T., Zhao Q., Mccully K.K. (2015). Measurement of Intramuscular Fat by Muscle Echo Intensity: Muscle Echo Intensity and Fat. Muscle Nerve.

[7] Ahtiainen J.P., Hoffren M., Hulmi J.J., Pietikäinen M., Mero A.A., Avela J., Häkkinen K. (2009). Panoramic Ultrasonography Is a Valid Method to Measure Changes in Skeletal Muscle Cross-Sectional Area. Eur. J. Appl. Physiol..

[8] Radaelli R., Botton C.E., Wilhelm E.N., Bottaro M., Lacerda F., Gaya A., Moraes K., Peruzzolo A., Brown L.E., Pinto R.S. (2013). Low- and High-Volume Strength Training Induces Similar Neuromuscular Improvements in Muscle Quality in Elderly Women. Exp. Gerontol..

[9] Rosenberg J.G., Ryan E.D., Sobolewski E.J., Scharville M.J., Thompson B.J., King G.E. (2014). Reliability of Panoramic Ultrasound Imaging to Simultaneously Examine Muscle Size and Quality of the Medial Gastrocnemius. Muscle Nerve.

[10] Scott J.M., Martin D.S., Ploutz-Snyder R., Caine T., Matz T., Arzeno N.M., Buxton R., Ploutz-Snyder L. (2012). Reliability and Validity of Panoramic Ultrasound for Muscle Quantification. Ultrasound Med. Biol..

[11] Carr J.C., Gerstner G.R., Voskuil C.C., Harden J.E., Dunnick D., Badillo K.M., Pagan J.I., Harmon K.K., Girts R.M., Beausejour J.P. (2021). The Influence of Sonographer Experience on Skeletal Muscle Image Acquisition and Analysis. JFMK.

[12] Hill J.C., Millán I.S. (2014). Validation of Musculoskeletal Ultrasound to Assess and Quantify Muscle Glycogen Content. A Novel Approach. Physician Sportsmed..

[13] Jenkins N.D.M. (2016). Are Resistance Training-Mediated Decreases in Ultrasound Echo Intensity Caused by Changes in Muscle Composition, or Is There an Alternative Explanation?. Ultrasound Med. Biol..

[14] Nieman D.C., Shanely R.A., Zwetsloot K.A., Meaney M.P., Farris G.E. (2015). Ultrasonic Assessment of Exercise-Induced Change in Skeletal Muscle Glycogen Content. BMC Sport. Sci. Med. Rehabil..

[15] Sarvazyan A., Tatarinov A., Sarvazyan N. (2005). Ultrasonic Assessment of Tissue Hydration Status. Ultrasonics.

[16] Akazawa N., Okawa N., Tamura K., Moriyama H. (2017). Relationships between Intramuscular Fat, Muscle Strength and Gait Independence in Older Women: A Cross-Sectional Study. Geriatr. Gerontol. Int..

[17] Isaka M., Sugimoto K., Yasunobe Y., Akasaka H., Fujimoto T., Kurinami H., Takeya Y., Yamamoto K., Rakugi H. (2019). The Usefulness of an Alternative Diagnostic Method for Sarcopenia Using Thickness and Echo Intensity of Lower Leg Muscles in Older Males. J. Am. Med. Dir. Assoc..

[18] Kawai H., Kera T., Hirayama R., Hirano H., Fujiwara Y., Ihara K., Kojima M., Obuchi S. (2018). Morphological and Qualitative Characteristics of the Quadriceps Muscle of Community-Dwelling Older Adults Based on Ultrasound Imaging: Classification Using Latent Class Analysis. Aging Clin. Exp. Res..

[19] Mirón Mombiela R., Facal de Castro F., Moreno P., Borras C. (2017). Ultrasonic Echo Intensity as a New Noninvasive In Vivo Biomarker of Frailty. J. Am. Geriatr. Soc..

[20] Yamada M., Kimura Y., Ishiyama D., Nishio N., Abe Y., Kakehi T., Fujimoto J., Tanaka T., Ohji S., Otobe Y. (2017). Differential Characteristics of Skeletal Muscle in Community-Dwelling Older Adults. J. Am. Med. Dir. Assoc..

[21] Bali A.U., Harmon K.K., Burton A.M., Phan D.C., Mercer N.E., Lawless N.W., Stock M.S. (2020). Muscle Strength, Not Age, Explains Unique Variance in Echo Intensity. Exp. Gerontol..

[22] Cadore E., Izquierdo M., Conceição M., Radaelli R., Pinto R., Baroni B., Vaz M., Alberton C., Pinto S., Cunha G. (2012). Echo Intensity Is Associated with Skeletal Muscle Power and Cardiovascular Performance in Elderly Men. Exp. Gerontol..

[23] Mota J.A., Stock M.S. (2017). Rectus Femoris Echo Intensity Correlates with Muscle Strength, but Not Endurance, in Younger and Older Men. Ultrasound Med. Biol..

[24] Stock M.S., Mota J.A., DeFranco R.N., Grue K.A., Jacobo A.U., Chung E., Moon J.R., DeFreitas J.M., Beck T.W. (2017). The Time Course of Short-Term Hypertrophy in the Absence of Eccentric Muscle Damage. Eur. J. Appl. Physiol..

[25] Dankel S.J., Abe T., Bell Z.W., Jessee M.B., Buckner S.L., Mattocks K.T., Mouser J.G., Loenneke J.P. (2020). The Impact of Ultrasound Probe Tilt on Muscle Thickness and Echo-Intensity: A Cross-Sectional Study. J. Clin. Densitom..

[26] Maughan R.J., Watson J.S., Weir J. (1983). Strength and Cross-Sectional Area of Human Skeletal Muscle. J. Physiol..

[27] Balshaw T.G., Massey G.J., Maden-Wilkinson T.M., Lanza M.B., Folland J.P. (2019). Neural Adaptations after 4 Years vs 12 Weeks of Resistance Training vs Untrained. Scand. J. Med. Sci. Sport..

[28] Stock M.S., Mota J.A., Hernandez J.M., Thompson B.J. (2017). Echo Intensity and Muscle Thickness as Predictors Of Athleticism and Isometric Strength in Middle-School Boys. Muscle Nerve.

[29] Stock M.S., Whitson M., Burton A.M., Dawson N.T., Sobolewski E.J., Thompson B.J. (2018). Echo Intensity Versus Muscle Function Correlations in Older Adults Are Influenced by Subcutaneous Fat Thickness. Ultrasound Med. Biol..

[30] Kleinberg C.R., Ryan E.D., Tweedell A.J., Barnette T.J., Wagoner C.W. (2016). Influence of Lower Extremity Muscle Size and Quality on Stair-Climb Performance in Career Firefighters. J. Strength Cond. Res..

[31] Mangine G.T., Hoffman J.R., Wang R., Gonzalez A.M., Townsend J.R., Wells A.J., Jajtner A.R., Beyer K.S., Boone C.H., Miramonti A.A. (2016). Resistance Training Intensity and Volume Affect Changes in Rate of Force Development in Resistance-Trained Men. Eur. J. Appl. Physiol..

[32] Schober P., Boer C., Schwarte L.A. (2018). Correlation Coefficients: Appropriate Use and Interpretation. Anesth. Analg..

[33] Song J.S., Abe T., Bell Z.W., Wong V., Spitz R.W., Yamada Y., Loenneke J.P. (2021). The Relationship Between Muscle Size and Strength Does Not Depend on Echo Intensity in Healthy Young Adults. J. Clin. Densitom..

[34] Watanabe Y., Yamada Y., Fukumoto Y., Ishihara T., Yokoyama K., Yoshida T., Miyake M., Yamagata E., Kimura M. (2013). Echo Intensity Obtained from Ultrasonography Images Reflecting Muscle Strength in Elderly Men. Clin. Interv. Aging.

[35] Buckner S.L., Yitzchaki N., Kataoka R., Vasenina E., Zhu W.G., Kuehne T.E., Loenneke J.P. (2021). Do Exercise-Induced Increases in Muscle Size Contribute to Strength in Resistance-Trained Individuals?. Clin. Physiol. Funct. Imaging.

[36] Wagle J.P., Carroll K.M., Cunanan A.J., Taber C.B., Wetmore A., Bingham G.E., DeWeese B.H., Sato K., Stuart C.A., Stone M.H. (2017). Comparison of the Relationship between Lying and Standing Ultrasonography Measures of Muscle Morphology with Isometric and Dynamic Force Production Capabilities. Sports.

[37] Wilhelm E.N., Rech A., Minozzo F., Botton C.E., Radaelli R., Teixeira B.C., Reischak-Oliveira A., Pinto R.S. (2014). Concurrent Strength and Endurance Training Exercise Sequence Does Not Affect Neuromuscular Adaptations in Older Men. Exp. Gerontol..

[38] Abe T., Kearns C., Fukunaga T. Sex Differences in Whole Body Skeletal Muscle Mass Measured by Magnetic Resonance Imaging and Its Distribution in Young Japanese Adults—PubMed. https://pubmed.ncbi.nlm.nih.gov/14514537/.

[39] Avin K., Naughton M., Ford B., Moore H., Monitto-Webber M., Stark A., Gentile A., Frey Law L. Sex Differences in Fatigue Resistance Are Muscle Group Dependent—PubMed. https://pubmed.ncbi.nlm.nih.gov/20195184/.

[40] Hicks A., Kent-Braun J., Ditor D. Sex Differences in Human Skeletal Muscle Fatigue—PubMed. https://pubmed.ncbi.nlm.nih.gov/11474957/.

[41] Shephard R., Bouhlel E., Vandewalle H., Monod H. Muscle Mass as a Factor Limiting Physical Work.—PMC. https://www-ncbi-nlm-nih-gov.ezproxy.tcu.edu/pmc/articles/PMC1197179/.

[42] Goodpaster B.H., He J., Watkins S., Kelley D.E. (2001). Skeletal Muscle Lipid Content and Insulin Resistance: Evidence for a Paradox in Endurance-Trained Athletes. J. Clin. Endocrinol. Metab..

[43] van Loon L.J.C., Goodpaster B.H. (2006). Increased Intramuscular Lipid Storage in the Insulin-Resistant and Endurance-Trained State. Pflug. Arch-Eur. J. Physiol..

[44] Pillen S., Tak R.O., Zwarts M.J., Lammens M.M.Y., Verrijp K.N., Arts I.M.P., van der Laak J.A., Hoogerbrugge P.M., van Engelen B.G.M., Verrips A. (2009). Skeletal Muscle Ultrasound: Correlation Between Fibrous Tissue and Echo Intensity. Ultrasound Med. Biol..

[45] Reimers K., Reimers C.D., Wagner S., Paetzke I., Pongratz D.E. (1993). Skeletal Muscle Sonography: A Correlative Study of Echogenicity and Morphology. J. Ultrasound Med..

[46] Katsiaras A., Newman A.B., Kriska A., Brach J., Krishnaswami S., Feingold E., Kritchevsky S.B., Li R., Harris T.B., Schwartz A. (2005). Skeletal Muscle Fatigue, Strength, and Quality in the Elderly: The Health ABC Study. J. Appl. Physiol..

[47] Melvin M.N., Smith-Ryan A.E., Wingfield H.L., Fultz S.N., Roelofs E.J. (2014). Evaluation of Muscle Quality Reliability and Racial Differences in Body Composition of Overweight Individuals. Ultrasound Med. Biol..

[48] Miljkovic I., Cauley J.A., Petit M.A., Ensrud K.E., Strotmeyer E., Sheu Y., Gordon C.L., Goodpaster B.H., Bunker C.H., Patrick A.L. (2009). Greater Adipose Tissue Infiltration in Skeletal Muscle among Older Men of African Ancestry. J. Clin. Endocrinol. Metab..

[49] Stock M.S., Oranchuk D.J., Burton A.M., Phan D.C. (2020). Age-, Sex-, and Region-Specific Differences in Skeletal Muscle Size and Quality. Appl. Physiol. Nutr. Metab..

[50] Liguori G., Feito Y., Fountaine C., Roy B. (2022). ACSM’s Guidelines for Exercise Testing and Prescription.

