# Supplementary material for: Skeletal Muscle Ultrasonography and Muscle Fitness Relationships: Effects of Scanning Plane and Echogenicity Correction
Source: Muscles. 2023 Mar 23;2(2):109–18. doi: 10.3390/muscles2020010 (PMC12225479; doi:10.3390/muscles2020010)
Supplement: Supplementary file 1 [file muscles-02-00010-s001.zip › muscles-2244808-supplementary.pdf]

Supplemental File S1

Prediction Model for 1RM Strength

1 RM Strength = −6.129 + 15.694(Sagittal Muscle Thickness) (2)

Table S1. Prediction Model for One Repetition Maximum

	Model			Descriptives of 1RM (lbs)		
	**B**	**SE of B**	**β**	**Mean**	**SD**	**N**
**Constant**	-6.129	7.884				
**Sagittal Muscle Thickness**	15.694*	2.429	0.836	43.87	11.96	20
**Model Summary**						
**R^2**	0.699					
**F**	41.742*					

B = unstandardized regression coefficient; SE of B = standard error of the coefficient
β = standardized coefficient; SD = standard deviation; N = sample size
R^2 = coefficient of determination
Significance at $p < 0.05$ denoted by *

Prediction Model for Total Repetitions

Total Repetitions = −45.641 + 0.640(Sagittal Corrected Echo Intensity) (3)

Table S2. Prediction Model for Total Repetitions

	Model			Descriptives of Repetitions		
	**B**	**SE of B**	**β**	**Mean**	**SD**	**N**
**Constant**	-45.641	25.249				
**Sagittal Corrected Echo Intensity**	0.640*	0.157	0.692	56.6	12.7	20
**Model Summary**						
**R^2**	0.478					
**F**	16.512*					

B = unstandardized regression coefficient; SE of B = standard error of the coefficient
β = standardized coefficient; SD = standard deviation; N = sample size
R^2 = coefficient of determination
Significance at $p < 0.05$ denoted by *
